# Additive Manufacturing of Smart Composite Structures Based on Flexinol Wires

**DOI:** 10.3390/ma15020499

**Published:** 2022-01-10

**Authors:** Olaf Dudek, Wojciech Klein, Damian Gąsiorek, Mariusz Pawlak

**Affiliations:** Faculty of Mechanical Engineering, Silesian University of Technology, ul. Konarskiego 18A, 44-100 Gliwice, Poland; olaf.dudek@polsl.pl (O.D.); wojciech.klein@polsl.pl (W.K.); damian.gasiorek@polsl.pl (D.G.)

**Keywords:** additive manufacturing, shape memory alloy, smart material, 4D printing

## Abstract

3D printing of a composite structure with shape memory materials requires a special approach to the subject, at the stage of the design and printing process. This paper presents the design steps during the development of a 3D-printed composite structure with shape memory material. The connection points between the SMA fibers and the printer filament are developed in the MATLAB environment. Finite element method is used to simulate the shortening of the shape memory material under the influence of temperature and its effect on the printed polymer material is presented. In the MATLAB environment, evolutionary algorithms were used to determine the shape of the SMA fiber alignment. This work demonstrates the use of shape memory effect in 3D printed smart composite structures, where the component takes a predetermined shape. The structure obtained as a result of such printing changes with the heat generated by the current voltage, making it the desired fourth dimension.

## 1. Introduction

The combination of 3D printing with smart materials to obtain predetermined deformation characteristics of the printed material is somewhat novel. The fact that it is possible to design the behavior of the material before it is printed, means that many new applications that were previously unavailable for the material can be found.

The beginnings of additive manufacturing technology date to the 1980s. The first 3D printing technology was stereolithography (SLA). Patent solutions and limitations of this technology led to the search for other printing techniques. Currently, it is possible to print from solid materials such as FDM (fused deposition modeling), powders such as SLS (selective laser sintering), DMLS (direct metal laser sintering), and CJP (colorjet printing); or liquid materials DLP (digital light processing), SLA (stereolithography), or POLYJET. Each of the printing techniques has its own applications, and knowing the limitations of these methods, users more consciously decide on their choice for selected applications. The search for new materials for 3D printing and the search for new possibilities of its application allowed the emergence of the concept of 4D printing in 2013. At the 2013 TEDConference, Tibbits [1,2] presented the concept of 4D printing, in which the printed model could change its shape over time. This effect was achieved by using smart materials in 3D printing. Smart materials react to external stimulation and can change their shape based on these stimuli [3,4,5]. An interesting study in the field of composites and intelligent polymers including the shape effect was presented in [6]. There is a growing number of works in the field of 4D printing, some of which provide an up-to-date review [7,8,9] on the potential and viability of 4DP. In the publications, information can be found about various smart materials and their activation mechanisms [10]. Interesting effects of using shape memory materials were presented in a paper [11], which showed the effect of shape change under stimuli and return to the original shape.

Shape memory alloys (SMA), thanks to their properties, are used in many fields of technology. Their history dates to the 1930s, when research began into pseudo-elasticity in Fe-Ni alloys. In 1963, William J. Buehler and Frederick Wang discovered the shape memory effect in Ni-Ti alloys, which was a milestone in this field of science. To this day, Ni-Ti alloys, commonly referred to as nitinol, are the most widely used alloys in technology.

SMA alloys belong to the group of intelligent materials and their characteristic feature is shape memory. The physical phenomenon responsible for this shape change is the martensitic transformation, which occurs as a result of a change in temperature. The change in shape—i.e., the deformation—reaches up to 15 percent for the monocrystalline structure and 8 percent for the polycrystalline structure [12,13]. As a result of the high level of reaction stress, reaching up to 500MPa, it is possible to restore the initial state with a significant load. A significant disadvantage of SMA alloys is the low frequency of transformation of the order of 102 Hz [14,15].

Due to the different processes and mechanisms that make up the shape memory effect, a distinction can be made [15]:

- pseudo-elasticity phenomenon;
- unidirectional shape memory effect;
- bi-directional shape memory effect.

**Figure 1:**
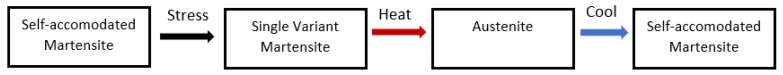
Demonstration of unidirectional shape memory effects.

In the Figure 1, there is a scheme of unidirectional shape memory effects. The heating of this component to the characteristic temperature causes the transformation of martensite to the austenite phase and restoration of the programmed shape. The structure after deformation returns to its original form in the temperature range from austenite start transformation, As, to austenite finish transformation, Af. The value of this deformation depends on the initially forced deformation [14,15].

The recovery of the original form of elements with unidirectional shape memory effect depends, to a large extent, on the number of transformation cycles [3,4]. As the transformation cycle increases and the amount of deformation increases, the ability to recover the original form decreases [14,15].

Alloys with a bidirectional shape memory effect have the ability to store the shape of the high-temperature parent phase and the low-temperature martensitic phase. To obtain this effect, the shape of the high-temperature parent phase and the low-temperature martensitic phase must be subjected to a repeated thermo-mechanical treatment, referred to as training.

Shape memory alloys are used, among other materials, as permanent mechanical and electrical connections, as heat engines, control systems, valve systems, vibration damping systems, actuation systems, diagnostic systems, and applications in biological organisms due to their biocompatibility with living tissues [16,17,18,19,20,21].

The shaping of actuators or sensors from SMA masters requires a complex process for their training. The solution to this problem can be the creation of any shape of actuators or sensors using incremental technologies. The use of preprogrammed SMA wires (Flexinol) in 3D printing will enable the achievement of the fourth dimension of printing. Such a print will be a composite consisting of a polymer matrix and SMA reinforcement. The freedom of geometry shaping in this technology will facilitate the implementation of SMA technology in various industries. The research on the application of the fourth dimension in cross-section technologies was carried out, among others, in [22].

Because SMA can achieve a 4D effect, the idea is to combine SMA with 3D printing FDM technology in a smart composite structure. The material used as a matrix is a material used in 3D printing processes. Copolyesters are a broad group of polymers ranging from naturally derived nonrenewable cellulosic polymers to synthetic copolymers PETg, PETG, PCTG, and PCTA. Chemically, copolyesters are obtained by reacting a polycondensation of an alcohol (in this case, ethylene glycol (EG) and/or cyclohexanodimethyl (CHDM)) with an acid (in this case, terephthalic acid (TPA) and/or isophthalic acid (IPA)).

Owing to various combinations and contributions of individual compounds, various polymers can be obtained with interesting property combinations and with different production costs [23].

The subject of the research is an intelligent composite structure consisting of a reinforcement in the form of Flexinol wires and a polymer matrix.

The research methodology described in this paper includes both numerical studies and preliminary experimental tests of the produced composite structures. The numerical research focused on the development of a simulation environment allowing for an appropriate selection of parameters of the designed structures (determination of the junctions between the Flexinol wires and the composite matrix). The tests, on the other hand, included the creation of structures using FDM printing technology and qualitative verification of the deformation of the structure after its activation.

## 2. Materials

Printed smart composite material described in this article consists of a polymer matrix, reinforcement in the form of a support fabric and an intelligent activator in the form of shape memory alloy fibers. The polymeric matrix should have sufficient stiffness to ensure that both the fibers and the composite can deform. The intelligent activator, applied as a fiber, made of shape memory alloy, deforms the structure through an external factor. The reinforcement is a load-bearing fabric, which carries the external loads generated by the composite element. As it was mentioned before, the material used as a matrix is a material used in 3D printing processes.

The first material used in the research was XT-CF20 [24], produced by the company ColorFabb located in Belfeld, the Nederlands, a plastic material based on Amphora™ 3D Polymer AM1800, enriched with carbon fibers, the amount of which is about 20%. The Amphora 3D material is manufactured by Eastman Chemical based in Kingsport, Tennessee in the United States and is based on copolyesters [25,26].

The second material is a metal alloy with shape memory (Smart Memory Alloy), popularly known as nitinol. Nitinol is a metallic alloy of nickel with titanium, where the approximate atomic percentage of the two elements is the same. It belongs to the group of smart materials exhibiting shape memory effect. The practical application is usually 53–57% nickel by weight [15].

The applied nitinol is in the form of a thin and slender fiber and bears the trade name Flexinol^®^ [27] and is manufactured by Dynalloy, Inc. located in Irvine, Canada. Flexinol actuators are wires with a small diameter dimension, programmed to contract into the shape of muscle fibers when the temperature is raised.

**Figure 2:**
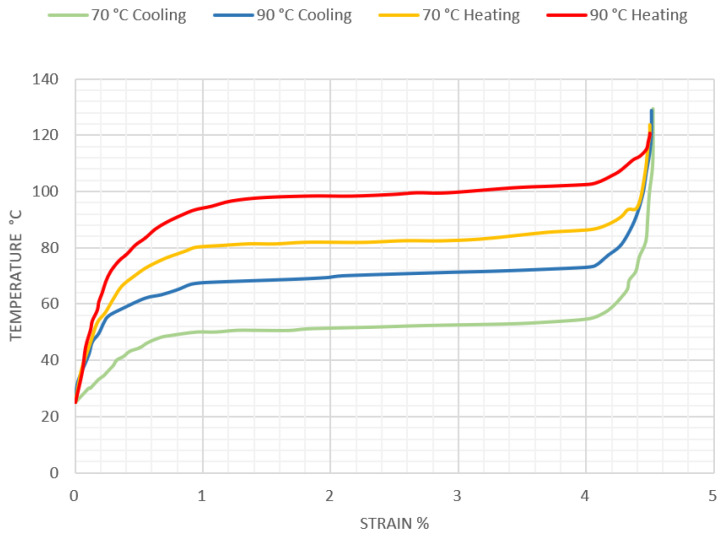
Temperature dependence of strain increase for Flexinol fibers.

The Flexinol fiber specification shows the relationship between melt temperature and generated strain. The pulling force of the fiber is constant. Figure 2 is generated from data available on Dynalloy.com [27], and shows this relationship for the two alloy types at 70 °C and 90 °C.

Flexinol was chosen for 4D printing due to the availability of different diameters and the possibility to choose the activation temperature. The paper does not focus on its microscopic properties. Instead, its macroscopic parameters such as force, activation temperature and geometric dimensions (diameter and length) were important elements. Thus, it was possible to produce composite structures by additive methods, which were shaped by fusing the Flexinol wires into the polymer matrix.

## 3. Method

### 3.1. Analytical Model

The basis for the complete execution and verification of numerical simulations is the development of a model and the performance of analytical calculations. Analytical calculations are aimed at determining the angle and deflection arrow of the bending beam, which is simplified to be a composite structure.

**Figure 3:**
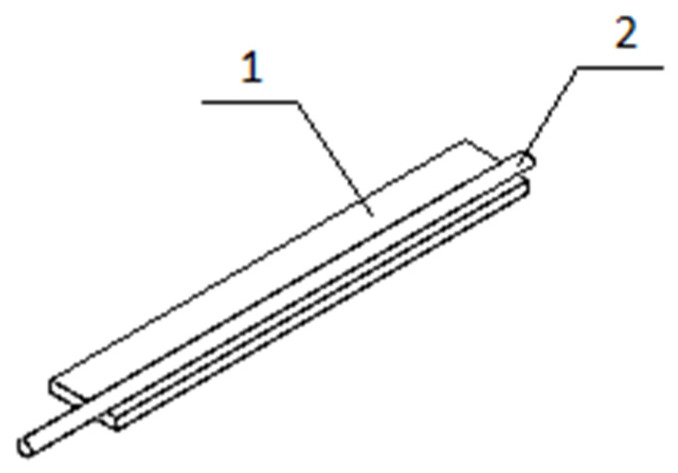
A fragment of a smart composite structure, wherein: 1—polymer 3D print ply, 2—SMA fiber.

The first step is to reduce the composite element to a single simple element representing the core of the phenomenon under investigation. The composite structure consists of individual actuators, which include one SMA fiber, which is the reinforcement and actuating element; and a matrix in the form of polymer, whose task is to stiffen the structure, keep the fibers in an appropriate position, and protect them from external factors. Thus, the example structure may consist of a finite number of beam elements. Figure 3 shows the top part of the material, detaching the SMA fiber.

**Figure 4:**
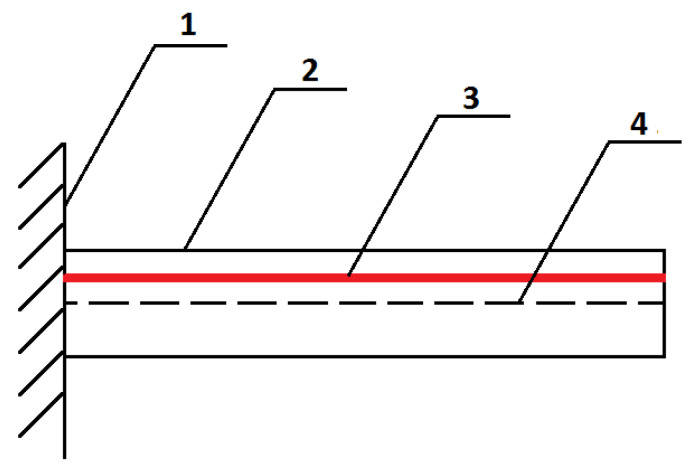
View of the beam element, where 1—fixed support of the beam; 2—beam; 3—SMA fiber; 4—beam axis of symmetry.

In order to consider the problem more easily, the system was reduced to a straight beam, which is the warp, while the SMA fiber inside the beam acts as the actuator, as shown in Figure 4.

**Figure 5:**
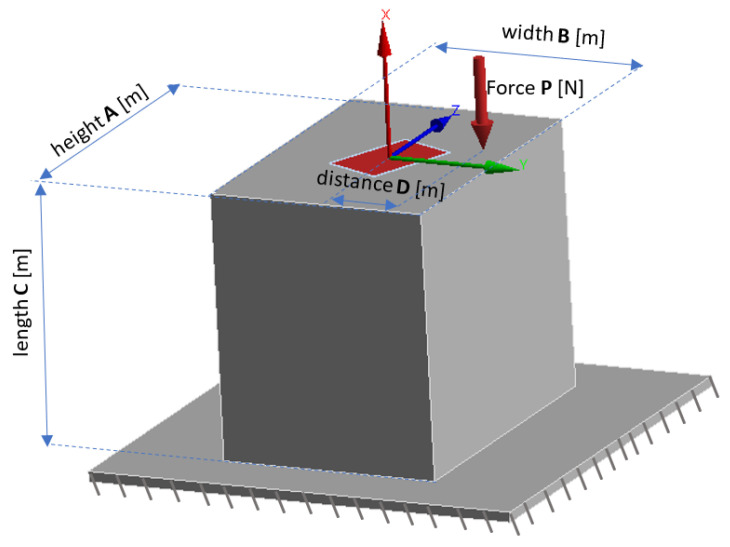
Finite beam element with visible beam cross-section.

The beam was split into a finite number of elements, where the SMA fiber as the actuator was replaced by a concentrated force applied at the cross-section, where the fiber runs parallel to the beam axis. This is shown in Figure 5.

Figure 5 shows the basic dimensions, i.e., the height of the beam is A and the width of the beam is marked as B. The distance C is the length of the finite element, while D is the distance of the SMA fiber and hence the force P from the center of the coordinate system. The coordinate system is located in the symmetry axis of the beam. The red color is the beam cross-section core.

The geometric location of all points of application of the force that induces stresses of the same sign in the whole section is called the cross-section core. The section core is the area in which a concentrated force can be applied without inducing stresses of the opposite sign due to a bending moment.

For the beam to bend, according to the deformation mechanism of beams or bars, the concentrated force must be applied outside the beam core area. If the concentrated force is applied inside the beam core, it only causes shearing of the beam because the fibers of the beam or bar are in compression throughout the cross-section, which means that only normal stresses occur.

**Figure 6:**
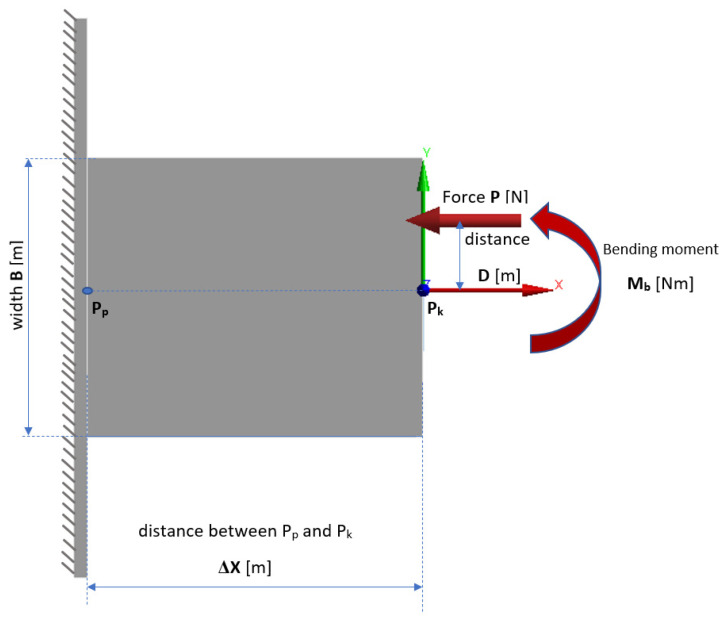
Simplified finite element model of a beam.

The basic issue in strength calculations of structures or their elements subjected to complex loading is load identification. Identification is the use of the laws of statics to determine the forces and moments acting on a structure or part of a structure from external loads. As a result, when a concentrated force is applied according to Figure 6 the load model is considered to be bending with shear, which is one of the cases of composite strength.

To derive a simplified analytical model and reduce the cost of numerical simulations, the deflection of an example composite structure was described as bending of beam elements, in a similar way as described in the literature [28]. The eccentric force, P, parallel to the x axis of the cantilever beam P_P_-P_k_ (Figure 6), is not applied at its centroid, but at a certain distance D from the neutral axis. It is a superposition of the compression or extension and bending conditions [29].

The eccentric force P is equivalent to two components, the axial force P (which is acting along the x axis) and the bending moment Mb (acting about the perpendicular z axis).

(1) $$M_{b}=P\;\times \;D$$

where M_b_—bending moment about the perpendicular axis (Nm); P—eccentric tensile force (N); D—distance from the neutral axis (m).

The deformations of the beam elements have been calculated according to the superposition method—i.e., the deformation of the beam under the influence of a compound load—is equal to the algebraic sum of the deformations caused by each of the loads acting separately. The maximum deflection of the cantilever beam is calculated from the equation

(2) $$\delta_{\mathrm{Pk}}=\frac{M_{b}\cdot ∆x^{2}}{2E\cdot I_{Z}}$$

where δ_Pk_—deflection of the beam in point P_k_ (m); ∆x—distance between point P_p_ and P_k_ (m); E—Elastic modulus (Pa); I_Z_—area moment of inertia (m^4^).

**Figure 7:**
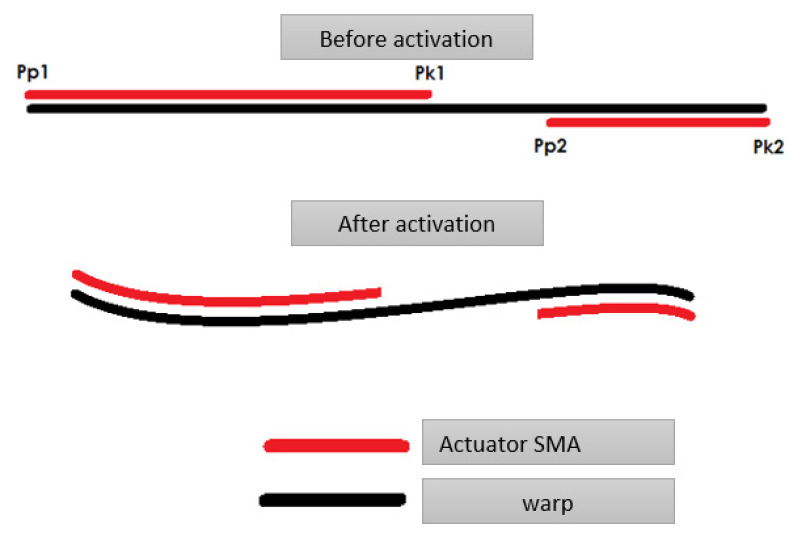
Shape of the beam before and after activation.

**Figure 8:**
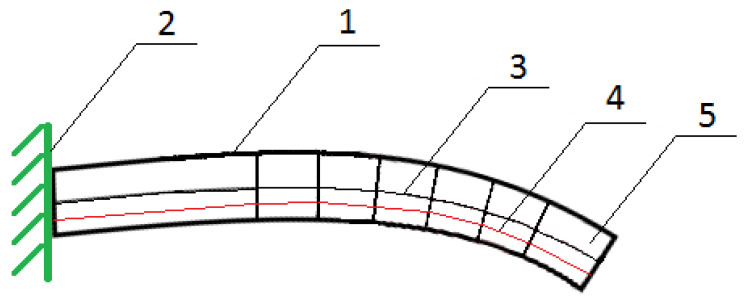
Schematic cross-section of a composite structure in the form of a bending beam, where: 1—bending beam; 2—fixed support of the beam; 3—neutral line during bending; 4—SMA fiber; 5—symmetry axis of the beam.

For the analytical calculations, a suitable model of a composite structure was adopted, which consists of a matrix and two fibers arranged along the beam axis, according to Figure 7 and Figure 8. The structure is considered as a beam element restrained on one side. The deformation force is generated by the shortening of the fibers, hence external forces act on the beam in each section, which can be compared to a continuous load distributed over the beam. The results of the analytical calculations for the solid structure are used for comparison with the experimental results. The structure is symmetrical, so in the second stage of the analytical calculations, for simplicity of calculation and comparison with simulation results, the focus was on the half structure with a single SMA activation fiber.

As a result of preliminary conceptual analyses, a shape memory alloy in the form of a wire with the trade name Flexinol was proposed as a smart activator. This alloy is a type of Nitinol, having a pre-programmed shape in the form of a straight wire. In the martensitic phase, high deformability is possible, which allows for arbitrary pathways in the composite structure. High strength and biocompatibility determine the use of this alloy. The activation mechanism is based on the supply of heat, under the influence of which the temperature of SMA fibers changes and the shape change mechanisms occur.

The heat can be supplied from the outside by heating or by passing an electric current through the SMA wire, which—due to its high resistance—heats up quickly and, as a result, changes shape. In addition, forcing with the flow of electric current allows easy control of the heating process by monitoring the parameters of its flow.

A thermoplastic was proposed as the matrix, due to the mechanism of formation of the composite shape change, the possibility of using it in available coordinate measuring machines for plastic processing under temperature, the easy availability of the material, and its low price. During activation with the use of electric current the SMA wire itself and a small part of the matrix in the vicinity are heated up, which favorably influences the forming mechanism of the whole composite element. The heated and plasticized matrix layer around the SMA wire gives freedom of movement between them. When the heat source is removed, the matrix cools and solidifies quickly, leaving the composite element in a deformed state. Activation by external heat supply heats up the matrix first, plasticizing it, and only then the SMA wire as an actuator, which is unfavorable due to the matrix plasticizing and flowing before the shaping process.

The purpose of the support fabric is both to carry the external loads during the life of the composite element and to be able to attach the SMA wire to it before covering it with the matrix layer. Figure 7 and Figure 8 show the geometry of the studied structure.

### 3.2. Numerical Model

**Figure 9:**
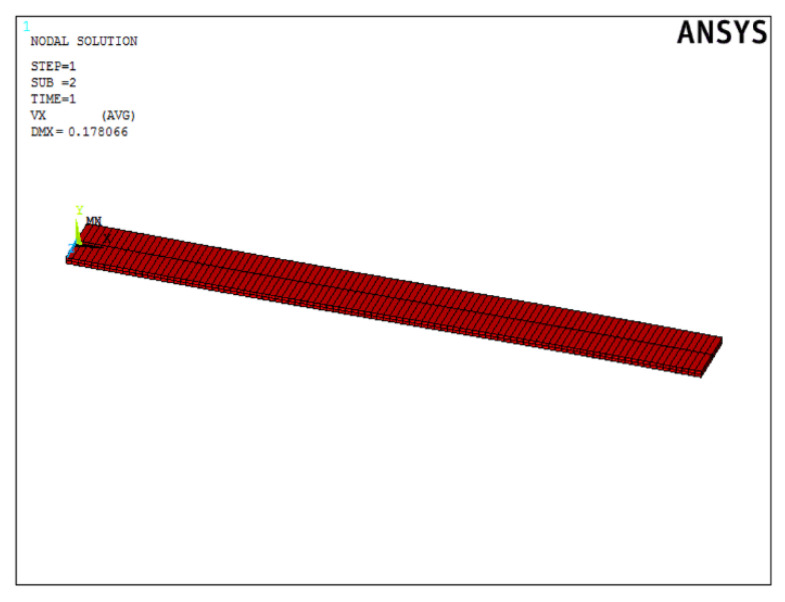
Beam structure in finite element method.

The model was developed in Ansys Mechanical environment by APDL macro, which can perform transient structural analysis using finite element method. The forcing was the temperature changing over time. The geometry of the modelled structure corresponds to the structure used in the analytical calculations. In this case, the structural model is a beam, fixed on one side, which consists of 100 one-dimensional beam elements. The size of each BEAM188 element is 1 mm, these elements are suitable for the analysis of slender and moderately thick beam structures. The element is based on Timoshenko beam theory, which includes shear effects. The element provides options for unrestricted warping and restrained warping of cross-sections. The element is a linear, quadratic, or cubic two-node beam element in 3D three-dimensional space. BEAM188 has six or seven degrees of freedom at each node. These include translations in the x, y, and z directions and rotations about the x, y, and z directions. The seventh degree of freedom (warping magnitude) is optional. The element is suitable for linear applications, large rotations and/or large nonlinear stresses. The element assumes stiffness and stress conditions, by default, in any analysis with large deviation. The supplied stiffness and stress conditions can analyze problems related to flexural, buckling, and torsional stability. The basic mechanisms of material models such as elasticity, plasticity, creep, and other non-linear material models are supported. The cross-section associated with this type of element can be a section of the development related to more than one material. Figure 9 shows a discretized form of a beam structure in the ANSYS APDL environment.

**Figure 10:**
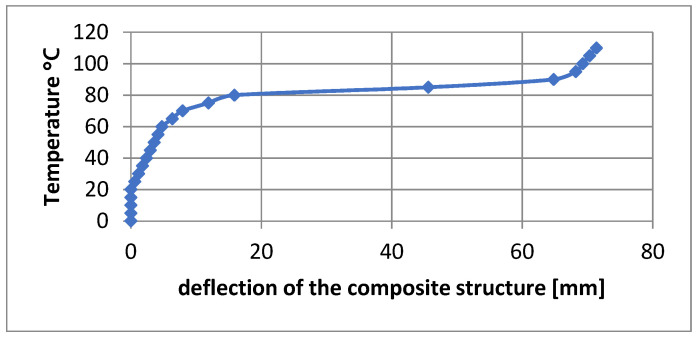
Temperature dependence of the deflection of the composite structure.

Effect of SMA fiber shortening as a function of temperature was implemented in simplified material model. This dependence was based on the graph presented in Figure 10, describing the increase in strain of the Flexinol Af 70 °C fiber as a function of temperature. For this purpose, a number of strength analyses of the composite structure in the form of a bent beam were carried out for different temperatures in the range from 0 to 110 °C, in 5 °C steps. The temperature, as a boundary condition, was set at the nodes of the beam. The structure was rigidly restrained at one end to correspond to clamping in a vice.

Twenty-three FEM numerical analyses were conducted to obtain information about the course of the deflection arrow depending on the given temperature. The course of deflection arrows of the composite structure is similar to the course of deformations depending on the same temperatures. Figure 10 shows the plot of the deflection arrow dependence of the composite structure on the given temperature.

Comparing the obtained curves of the beam deflection as a function of temperature, it can be stated that it is identical to the course of the SMA material deformation as a function of temperature. The effect of SMA fiber shortening as a function of temperature was applied in the material model by negative thermal expansion coefficient. Such a simplification in FEM analysis is aimed at reducing the solution of problems with non-linearity of deformations. As a result, the calculation time is significantly reduced, which is an important condition for further work.

The main objective of the numerical simulation was to identify the geometric parameters of the analyzed composite structure, related to the SMA fibers length and position. In identification process evolutionary algorithms from MATLAB toolbox were applied [30], where the fitness function from each iteration was compared. The MAC (modal assurance criterion) was used to determine the degree of agreement between the shape of the reference pattern and the actual nodal solution [31,32,33].

(3) $$\mathrm{MAC}\;\left(\mathrm{sp},\mathrm{ns}\right)=\frac{{\left|{\left\{\varphi_{\mathrm{sp}}\right\}}_{\;}^{T}\left\{\varphi_{\mathrm{ns}}\right\}\right|}^{2}}{\left({\left\{\varphi_{\mathrm{sp}}\right\}}_{\;}^{T}\left\{\varphi_{\mathrm{sp}}\right\}\right)\left({\left\{\varphi_{\mathrm{ns}}\right\}}_{\;}^{T}\left\{\varphi_{\mathrm{ns}}\right\}\right)},\;$$

where: MAC—Modal Assurance Criterion; φ_sp_—vector of shape pattern nodes; φ_ns_—vector of actual nodal solution.

Ansys Mechanical solver was used to run transient structural analysis, based on finite element method, and to transfer results in matrix form to specified files.

**Figure 11:**
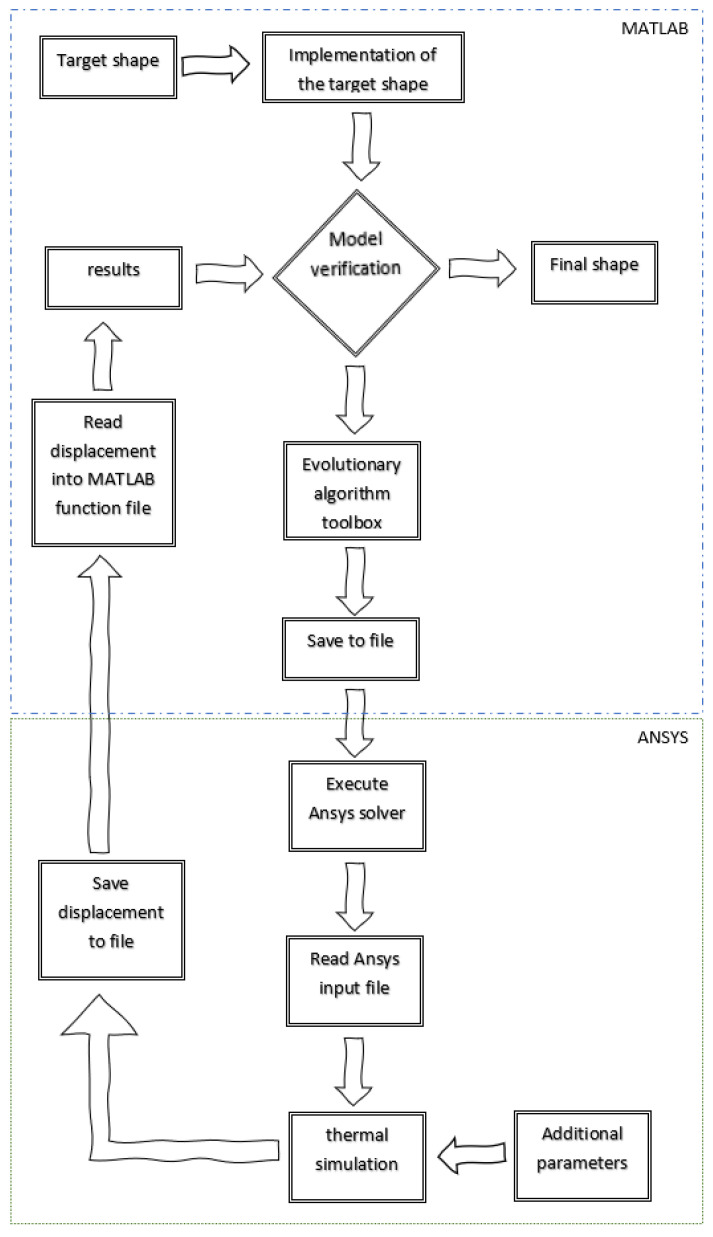
Diagram of the optimization.

MATLAB was responsible for reading results from Ansys files, running evolutionary algorithms with specified objective functions and identifying geometry parameters. The steps of the optimization process are presented in Figure 11.

The first step of the algorithm is to enter the physical data and material data of the tested composite into the program executed by the ANSYS environment, used as a solver. It is necessary to determine the dimensions of the composite and the cross-sections of the matrix and reinforcement in the form of SMA. The material data of the matrix and reinforcement are then entered into the solver and the type of beam element is specified. Once the geometry and material properties have been assigned, the boundary conditions are entered in the form of the withdrawal of degrees of freedom of the individual nodes and temperature functions. These are the input data required to perform the FE analysis of the composite structure.

Next, the pattern—i.e., the target shape of the structure—is generated and then implemented in the MATLAB environment. Such a pattern is a set of points in the form of a matrix with coordinates of points.

In the next step, verification occurs. The verification is expressed by the objective function, which in turn is expressed by the MAC criterion. This criterion compares the current form to the pattern and returns a value from 0 to 1, where 0 indicates a definite lack of conformity, while 1 means full conformity to the pattern. If the verification is successful (YES), the result is returned and the fibers are arranged in a way that allows the composite shape to be made. If the verification is negative (NO), the algorithm continues and moves to the genetic algorithm section. The genetic algorithm has the task of selecting an appropriate arrangement of SMA fibers. In the first phase, the algorithm selects a population—i.e., a number of random string arrangements. In the next phase, completely negative or wrong fibers are discarded, while the positive ones are passed on and slightly modified.

After selecting the arrangement of the wires, a file is saved as the coordinates of the structure points and a command is issued which runs the program in the ANSYS environment, where the file is loaded. Then, the solver is run in the form of an FEA strength analysis. The model of the composite structure is subjected to thermal loading. After the analysis is completed, the deformed form is written to the file as a set of coordinates of the structure points. In a MATLAB environment, the model is read from the file and subjected to further verification.

## 4. Results

In a first step, test simulations were carried out to select the appropriate parameters of the genetic algorithm to obtain correct results in a short time. Then, a series of simulations were carried out for three patterns of deformed beam.

### 4.1. Sensitivity Analysis

**Figure 12:**
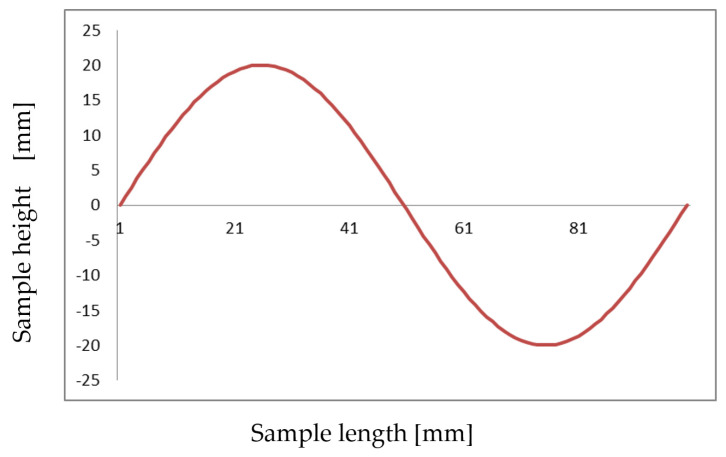
Exemplary target shape.

Figure 12 shows the shape of the test pattern that was used to carry out the sensitivity analysis.

Table 1 shows the parameters of the test simulations and the value of the objective function together with the results.

**Figure 13:**
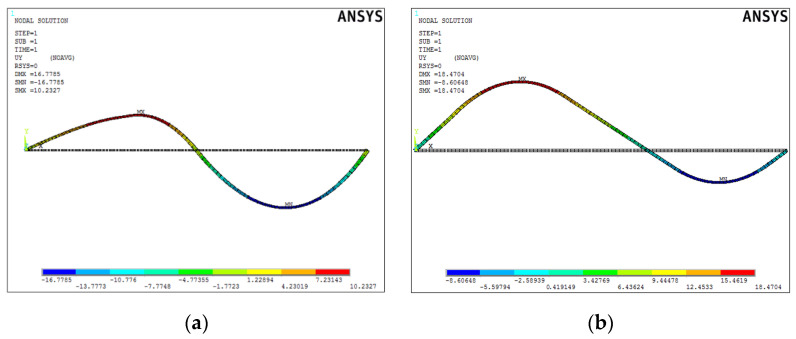
Obtained UY displacement distribution of the beams (in mm) after FEM analysis: (**a**) for simulation 1, (**b**) for simulation 2.

**Figure 14:**
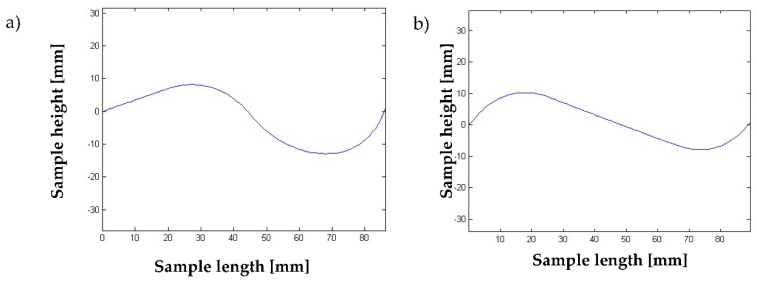
Obtained displacement distribution (in MATLAB) of the beams after FEM analysis: (**a**) for simulation 1, (**b**) for simulation 2.

**Figure 15:**
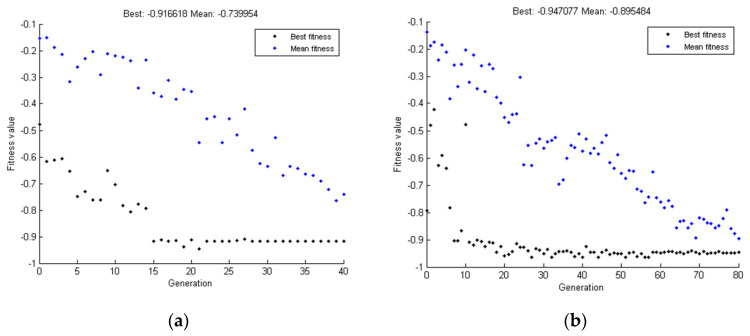
Mean and maximum value of the objective function depending on the individual generations: (**a**) for simulation 1, (**b**) for simulation 2.

Below (Figure 13, Figure 14 and Figure 15), the results of two test simulations are presented for population size 10 and number of generations equal to 40, and for population size 100 and number of generations equal to 80. The simulations were based on a test pattern and their aim was to check what number of individuals and generations would be sufficient to arrive at the best solution, and to verify the process of finding the best solution by the genetic algorithm.

Based on the above results, it was found that a larger number of populations and generations increases the accuracy of the obtained results, but also increases the calculation time by a factor of two. In both simulations, the value of the objective function did not exceed the limit after which the algorithm considers the obtained result as the best. The value of the objective function must agree 98.5% with the pattern, then the algorithm will stop and return the best result.

### 4.2. Shape Pattern ‘Sinus’ Simulation

**Figure 16:**
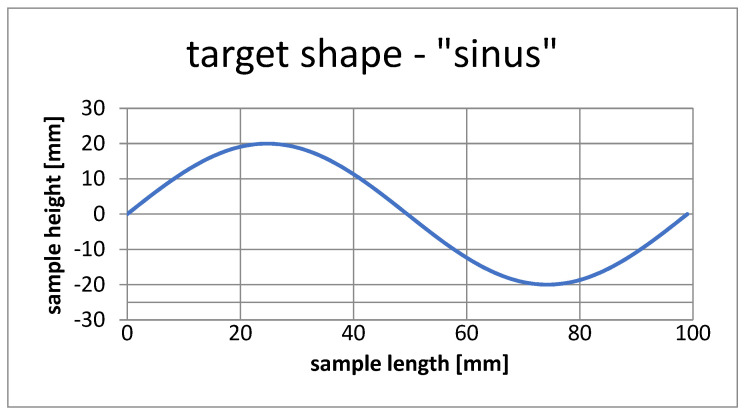
Target shape, case number 1.

Figure 16 shows the template used to simulate the selection of SMA fiber placement in the composite structure to achieve the given shape.

**Figure 17:**
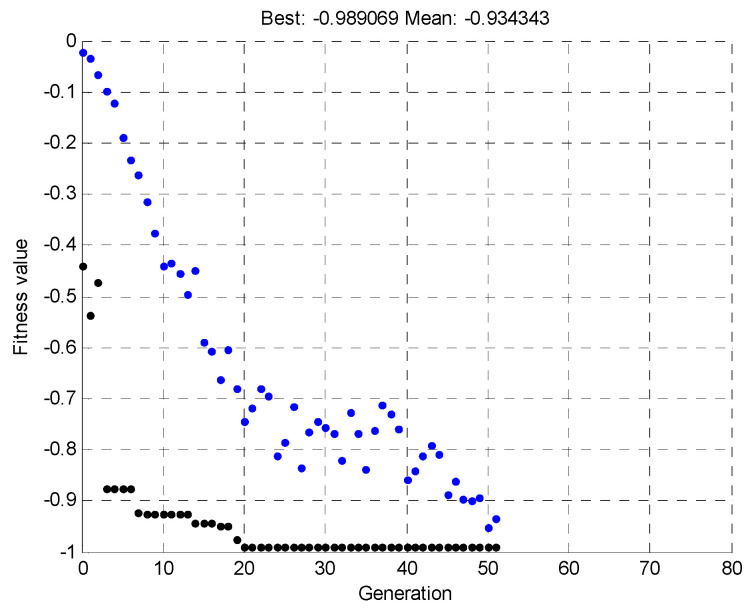
Mean and maximum values of the objective function depending on the individual generations for the first simulation.

Once the pattern was determined, a simulation was run, for 100 individuals and 80 generations. The simulation was terminated before the specified time due to the early achievement of the best solution, within the tolerance range of the objective function values. Figure 17 shows the mean and maximum value of the objective function, depending on the individual generations.

**Figure 18:**
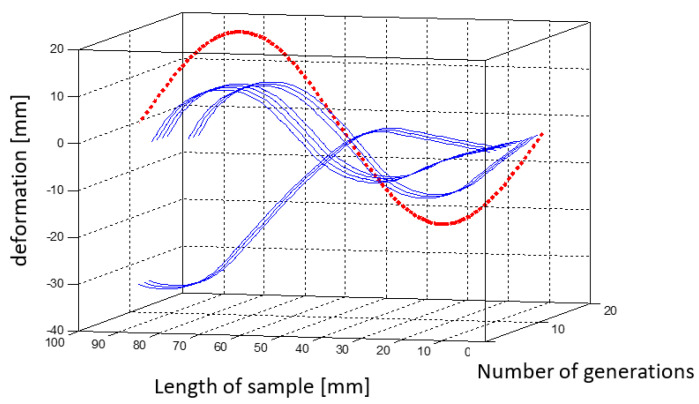
Graph showing changes in the shape of the element with successive iterations of the first simulation.

Figure 18 is a three-dimensional plot that shows the changing shape of the element with successive iterations of the simulation.

**Figure 19:**
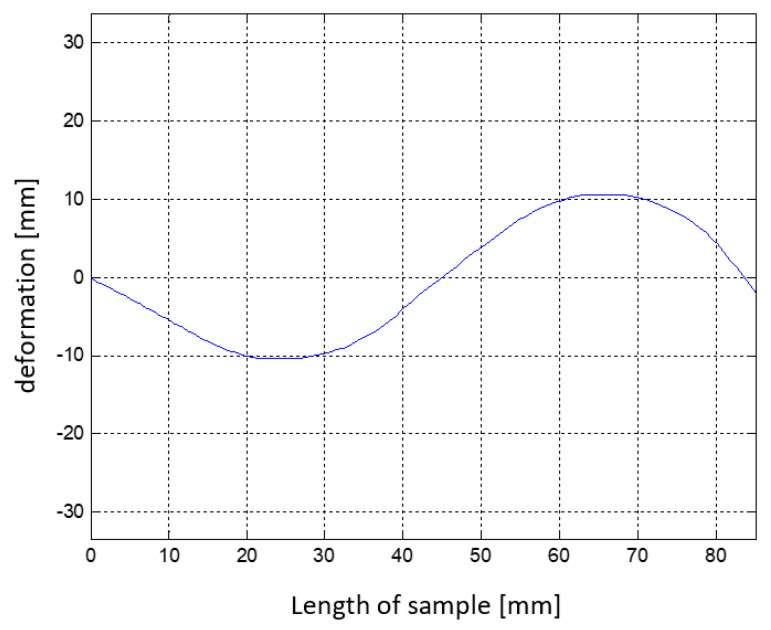
Dimensions of received sample from first simulation.

Figure 19 shows the best of the sample forms obtained from optimization.

Table 2 shows the parameters of the simulations and the value of the objective function together with the results.

**Figure 20:**
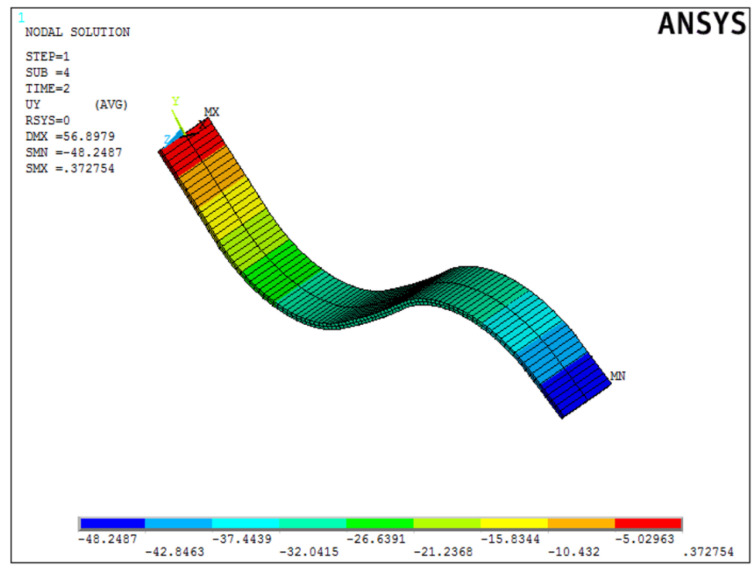
Displacement distribution of the sample form from the first simulation in mm.

Once the best solution was obtained, it was verified in the ANSYS APDL environment. Figure 20 shows the deformed form of the beam.

### 4.3. Shape Pattern ‘U’ Simulation

**Figure 21:**
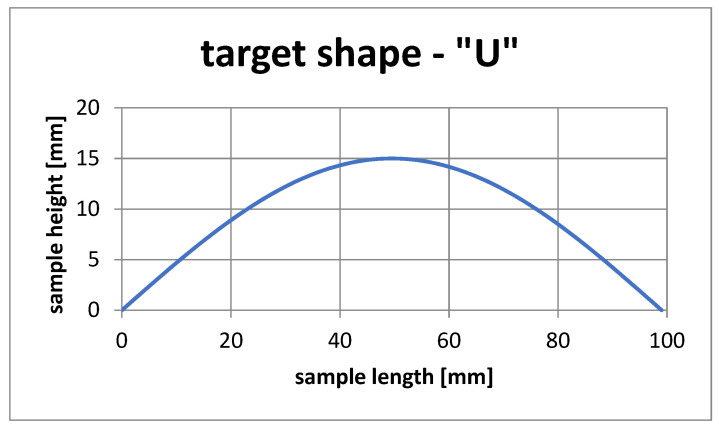
Target shape, case number 2.

Figure 21 shows the template used to simulate the selection of SMA fiber placement in the composite structure to achieve the given shape.

**Figure 22:**
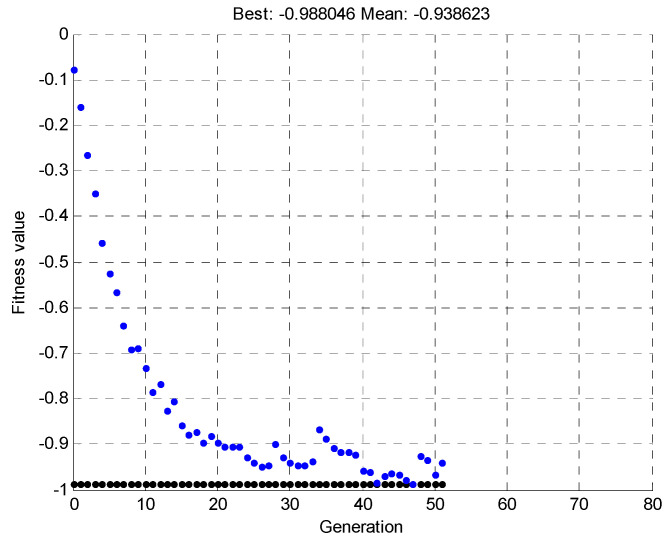
Mean and maximum value of the objective function as a function of the individual generations for the second simulation.

Once the pattern was determined, a simulation was run, for 100 individuals and 80 generations. The simulation was terminated before the specified time due to the early reaching of the best solution, within the tolerance range of the objective function values. Figure 22 shows the mean and maximum value of the objective function, depending on the individual generations.

**Figure 23:**
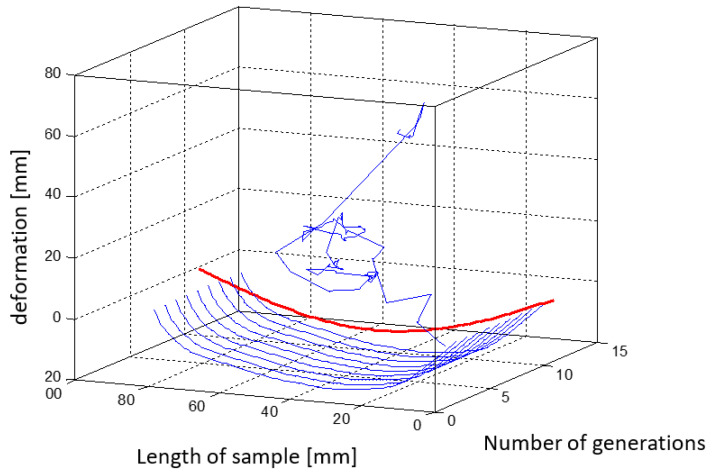
Graph showing changes in the shape of the element with successive iterations of the second simulation.

Figure 23 shows a three-dimensional plot that shows the changing shape of the element with successive iterations of the simulation.

**Figure 24:**
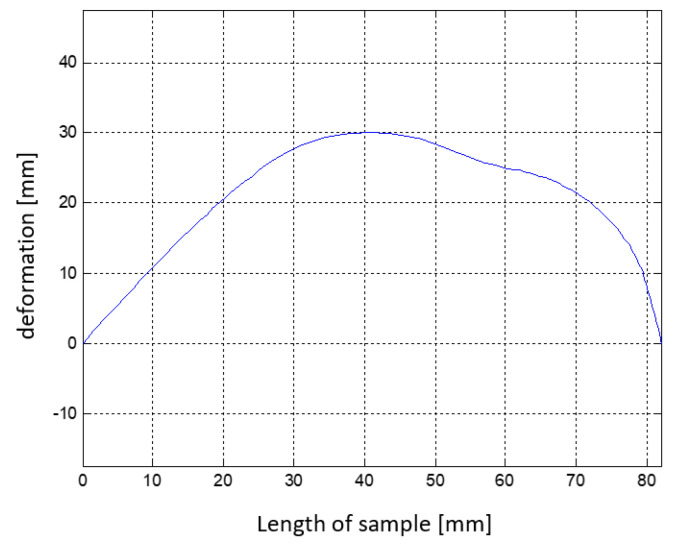
Best of the sample forms.

**Figure 25:**
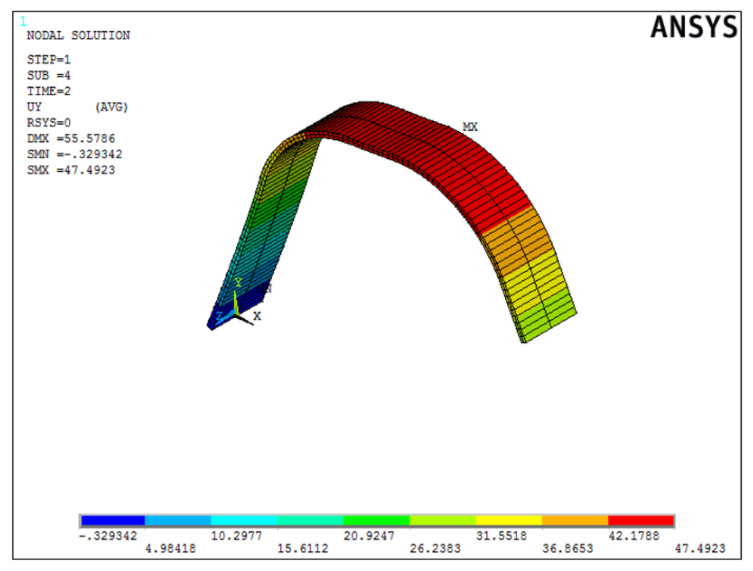
Displacement distribution of the sample form from the second simulation in mm.

**Figure 26:**
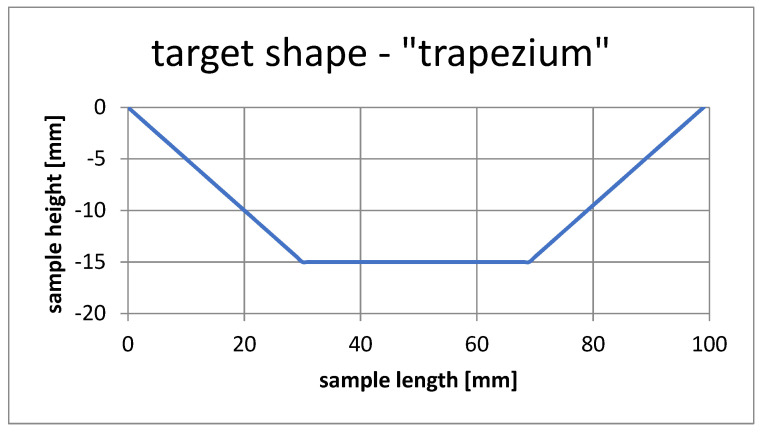
Target shape, case number 3.

**Figure 27:**
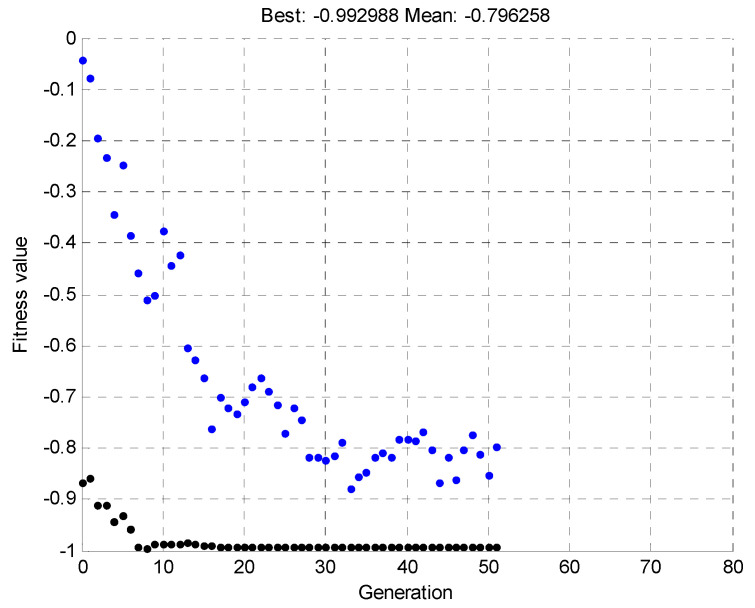
Mean and maximum values of the objective function as a function of the individual generations for the third simulation.

**Figure 28:**
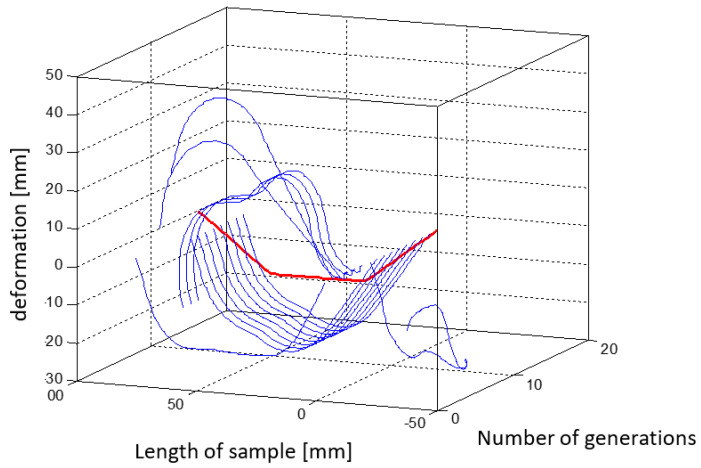
Graph showing changes in the shape of the element with successive iterations of the third-party simulation.

The random lines from the first iteration (presented in Figure 23, Figure 24, Figure 25, Figure 26, Figure 27 and Figure 28) are results of the random nature of the decision variables selection in the genetic algorithm. Constraints were general and did not protect the structure against forbidden arrangement of wires—e.g., two wires located in one place. In further iterations of the genetic algorithm, the objective function promotes individuals with the physical deflection capability of the beam to match its target shape.

In the simulations, genetic algorithms were implemented in the MATLAB GA genetic algorithm toolbox. The algorithm stops if the average relative change in the best fitness function value over generations is less than or equal to 1 × 10^−4^. Figure 24 shows the results from 52 iterations of the genetic algorithm. According to Figure 22, the mean and maximum values are tested, and the best in a given generation (out of 100 individuals in a generation) has a chance of crossover. The algorithm minimizes the objective function MAC with a minus sign.

Figure 24 shows the best of the sample forms obtained from optimization process.

Table 3 shows the parameters of the simulations and the value of the objective function together with the results.

Once the best solution was obtained, it was verified in the ANSYS APDL environment. Figure 25 shows the deformed form of the beam.

### 4.4. Shape Pattern “Trapezium” Simulation

Figure 26 shows the template used to simulate the selection of SMA fiber placement in the composite structure to achieve the given shape.

Once the pattern was determined, a simulation was run, for 100 individuals and 80 generations. The simulation was terminated before the specified time due to the early achievement of the best solution, within the tolerance range of the objective function values. Figure 27 shows the mean and maximum value of the objective function, depending on the individual generations.

Figure 28 shows a three-dimensional plot that shows the changing shape of the element with successive iterations of the simulation.

**Figure 29:**
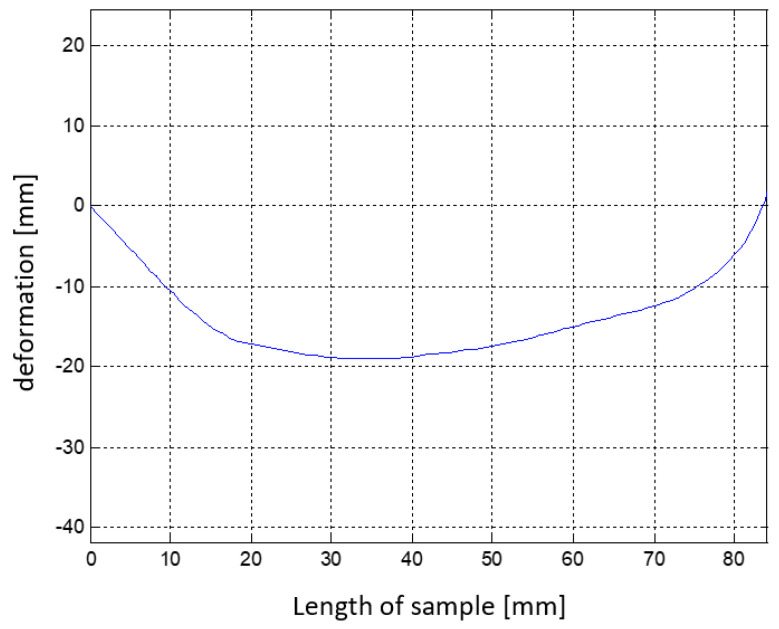
This is a figure. Schemes follow the same formatting.

Figure 29 shows the best of the sample forms obtained from optimization process.

Table 4 shows the simulation parameters and the value of the objective function with together with the results.

**Figure 30:**
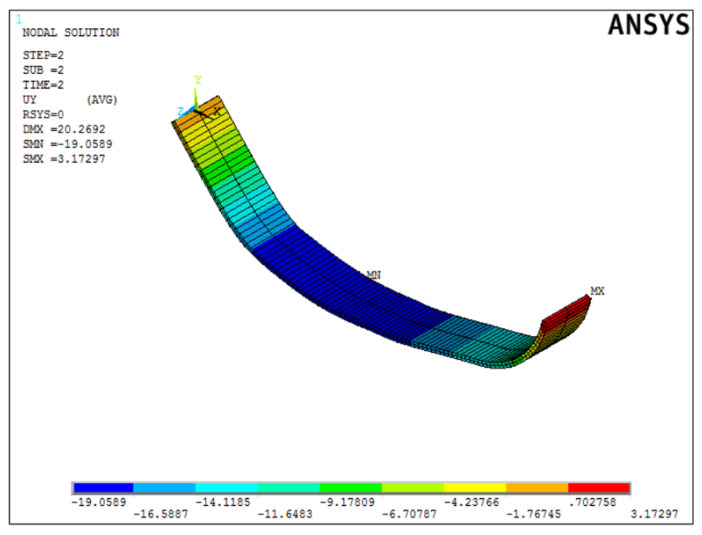
Displacement distribution of the sample form from the third simulation in mm.

Once the best solution was obtained, it was verified in the ANSYS APDL environment. Figure 30 shows the deformed form of the beam.

Function files created in MATLAB, and APDL files created in Ansys Mechanical, allow parameterization of the geometric model and optimization of the entire system. A qualitative analysis was carried out, the example of a 4D FDM print with SMA material presented in Section 5 is the result of numerical simulations and optimizations presented in this section. With the shape pattern U the actuator produced according to these geometrical parameters meets the assumptions made in numerical simulations.

To simplify calculations, the focus was on half of the structure with a single SMA activation fiber. Comparing the obtained maximum beam deflection as a function of temperature, it can be stated that it is identical to the course of the SMA material deformation as a function of temperature. Such a simplification in FEM analysis was aimed at reducing the solution of problems of non-linearity of deformations. As a result, the time of the performed calculations was significantly reduced, which is an important condition for further work.

## 5. Sample of Actuator

**Figure 31:**
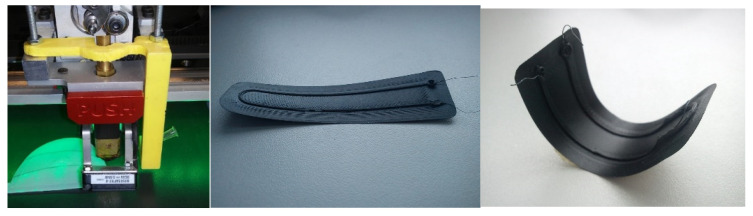
3D printer head with additional nitinol applicator, samples of an actuator made by 3D printing.

In the phase of layered FDM 3D printing of filaments with SMA fibers, the focus was on a way to produce a composite with the complete use of a coordinate device, using a 3D printer as an example. The method consists of printing a layer of matrix flake, fusing the SMA wire using a second head and covering the path with the matrix layer. Activation of the composite took place via the flow of electric current of 200 mA, which effectively raised the internal temperature of the wire within 5 s. As the wire was heated, the matrix was also heated, which after softening allowed the SMA wire to deform. When the electric current source was captured, the solidifying matrix bound the SMA wire in the form of a deformed composite. The Figure 31 shows a composite sample before and after activation. 

**Figure 32:**
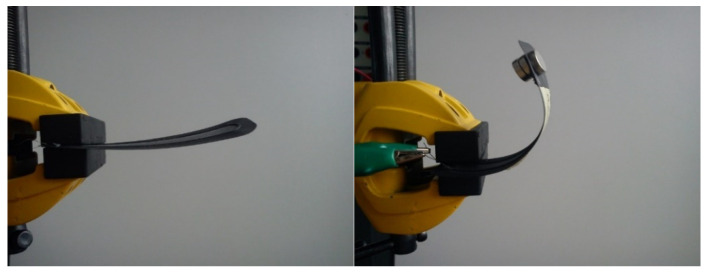
3D printed actuator sample, clamped in a vice.

On Figure 32, the final bending angle of the edge of the actuator is approximately 180°. The composite was also tested with an approximately 10-g load of three neodymium magnets. The sample with additional load deformed more slowly. The same specimen was tested several times, which caused the SMA wire to melt through the matrix. The phenomenon can be referred to as specimen failure.

**Figure 33:**
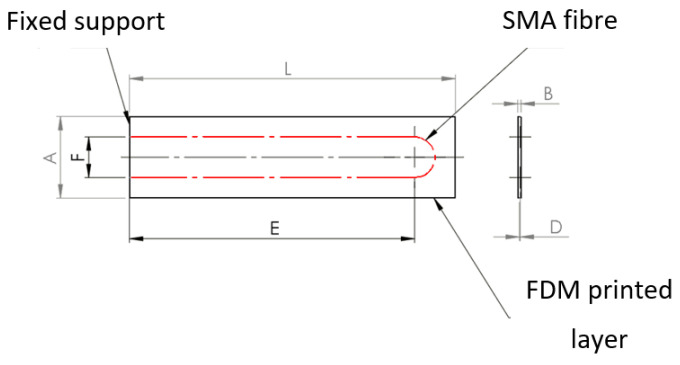
Geometrical form of the tested composite structure.

Considering the assumptions of automation and simplicity of the process and the use of inexpensive and accurate methods, the method of layered 3D printing (Figure 33) is the most promising. However, due to the uniqueness of effects in sequentially created samples, it is necessary to verify the method of SMA wire application and theoretical and practical understanding of the deformation mechanism. It is also necessary to carry out numerical analyses on different variants of the specimens to verify the positions of the neutral planes and thus exclude random deformations.

## 6. Conclusions

The paper presents innovative technology for shaping smart composites by using the shape memory phenomenon in fibers with the trade name Flexinol. For this purpose, an experimental verification of technological possibilities of manufacturing intelligent composite structures was carried out, as well as simulation studies in MATLAB and ANSYS software. The designed composite structure deforms according to assumptions, and the direction of beam deflection depends on the position of the SMA fiber in relation to the bending neutral axis.

The process of identification of geometric parameters of SMA fiber arrangement was realized by means of genetic algorithms. The convergence of this process in the determination of the given shape can be seen by minimizing the proposed objective function.

Due to the introduction of simplifications in the numerical model, the time required to perform the necessary calculations—which was about 4 h for a single case—was reduced.

The methods of intelligent shaping of composite structures presented in this paper are very promising and further research will be applied. Already, some improvements in the proposed technology were found. One of them is to carry out work to check the possibility of obtaining a more accurate reproduction of the shape of the pattern by using more SMA fibers. This is associated with an increase in the total of variables in the numerical model, which will have an impact on the time of carrying out every single simulation. It is also worth checking if there are already developed discrete models which are more appropriate for mapping spatial shapes.

Another option is to carry out experimental tests with accurate measurements, to compare experimental data with the results of numerical simulations, and improve the technology of production of such structures. A major challenge is the accurate alignment of the SMA fibers with respect to the neutral axis in the bending beam, as this is a parameter that significantly affects the deflection of the beam. An answer to this production task may be the use of additive printing technology in combination with composite lamination technology.

The research is preliminary and requires further development, particularly on the experimental side. Authors plan to develop a dedicated measuring stand, which will allow the measurement of deformations in the structure in three axes. Thanks to these measurements, it will be possible to correlate experimental results with the obtained numerical results, which will make it possible in the future to develop advanced algorithms for the selection of parameters of this class of intelligent structures.

## 7. Patents

Patents in Poland (in polish) resulting from the work reported in this manuscript:

PL 230 849 B1 Sposób wytwarzania inteligentnych rdzeni dla wysokowytrzymałych struktur kompozytowych 1.12.2018 WUP 12/18.

PL 230 850 B1 Sposób kształtowania inteligentnych struktur kompozytowych 31.12.2018 WUP 12/18.

PL 231 574 B1 Sposób wytwarzania inteligentnych struktur kompozytowych metodą druku 3D 29.03.2019 WUP 03/19.

## Figures and Tables

**Table 1:** Test simulation parameters and the value of the objective function together with the results.

Simulation number	1	2
Population size	10	100
Number of generations	40	80
Value of objective function	0.92	0.95
Point Pp1	31	22
Point Pk1	44	38
Point Pp2	61	71
Point Pk2	84	87

**Table 2:** First simulation parameters and the value of the objective function together with the results.

Simulation Variant	‘Sinus’
Population size	100
Number of generations	80
Generation with the best solution	52
Value of objective function	0.989
Point Pp1	17
Point Pk1	44
Point Pp2	63
Point Pk2	93

**Table 3:** Second simulation parameters and the value of the objective function together with the results.

Simulation Variant	‘U’
Population size	100
Number of generations	80
Generation with the best solution	52
Value of objective function	0.988
Point Pp1	78
Point Pk1	100
Point Pp2	28
Point Pk2	54

**Table 4:** Test simulation parameters and the value of the objective function together with the results.

Simulation Variant	“Trapezium”
Population size	100
Number of generations	80
Generation with the best solution	52
Value of objective function	0.993
Point Pp1	33
Point Pk1	56
Point Pp2	66
Point Pk2	90

## Data Availability

The data presented in this study are available on request from the corresponding author.
